# Natural killer cell-related prognosis signature characterizes immune landscape and predicts prognosis of HNSCC

**DOI:** 10.3389/fimmu.2022.1018685

**Published:** 2022-10-03

**Authors:** Hao Chi, Xixi Xie, Yingjie Yan, Gaoge Peng, Dorothee Franziska Strohmer, Guichuan Lai, Songyun Zhao, Zhijia Xia, Gang Tian

**Affiliations:** ^1^ Clinical Medical College, Southwest Medical University, Luzhou, China; ^2^ School of Stomatology, Southwest Medical University, Luzhou, China; ^3^ Department of Plastic and Reconstructive Surgery, Shanghai Ninth People’s Hospital Affiliated to Shanghai Jiaotong University School of Medicine, Shanghai, China; ^4^ Department of General, Visceral, and Transplant Surgery, Ludwig-Maximilians-University Munich, Munich, Germany; ^5^ Department of Epidemiology and Health Statistics, School of Public Health, Chongqing Medical University, Chongqing, China; ^6^ Department of Neurosurgery, Wuxi People's Hospital Affiliated to Nanjing Medical University, Wuxi, China; ^7^ Department of Laboratory Medicine, The Affiliated Hospital of Southwest Medical University, Luzhou, China

**Keywords:** natural killer cell, HNSCC, tumor microenvironment, immunotherapy, prognostic signature

## Abstract

**Background:**

Head and neck squamous cell carcinoma (HNSCC), the most common head and neck cancer, is highly aggressive and heterogeneous, resulting in variable prognoses and immunotherapeutic outcomes. Natural killer (NK) cells play essential roles in malignancies’ development, diagnosis, and prognosis. The purpose of this study was to establish a reliable signature based on genes related to NK cells (NRGs), thus providing a new perspective for assessing immunotherapy response and prognosis of HNSCC patients.

**Methods:**

In this study, NRGs were used to classify HNSCC from the TCGA-HNSCC and GEO cohorts. The genes were evaluated using univariate cox regression analysis based on the differential analysis of normal and tumor samples in TCGA-HNSCC conducted using the “limma” R package. Thereafter, we built prognostic gene signatures using LASSO-COX analysis. External validation was carried out in the GSE41613 cohort. Immunity analysis based on NRGs was performed *via* several methods, such as CIBERSORT, and immunotherapy response was evaluated by TIP portal website.

**Results:**

With the TCGA-HNSCC data, we established a nomogram based on the 17-NRGs signature and a variety of clinicopathological characteristics. The low-risk group exhibited a better effect when it came to immunotherapy.

**Conclusions:**

17-NRGs signature and nomograms demonstrate excellent predictive performance and offer new perspectives for assessing pre-immune efficacy, which will facilitate future precision immuno-oncology research.

## Introduction

HNSCC accounts for 95% of head and neck cancers and causes over 316,000 annual deaths worldwide (1, 2). Since the head and neck region is home to many vital organs that control important physiological functions, and a large number of muscles, bones, blood vessels and nerves are concentrated in a rather small space with interlocking organ sites (3), traditional treatment methods such as surgery, chemotherapy and radiotherapy are difficult to eradicate the disease. Compared with traditional methods, NK immune cells are more precisely targeted and can achieve the effect of removing cancer cells, anti-relapse and anti-metastasis. However, due to the highly aggressive and heterogeneous nature of HNSCC, the prognosis of patients remains poor, with morbidity and mortality rates increasing year by year (4). The TNM stage and histological grade are closely correlated with the prognosis of HNSCC and are also the main basis and foundation for treatment options such as prognostic grading, immunotherapy, radiation and chemotherapy (5). HNSCC patients may, however, demonstrate different clinicopathologic characteristics, suggesting that traditional clinicopathologic staging may not be completely accurate (6). Consequently, finding new prognostic biomarkers is crucial to improving the quality of life of HNSCC patients.

NK cells, defined as CD3-CD16+CD56+ lymphocytes, are the third class of lymphocytes in the body, accounting for approximately 5% to 15% of the circulating lymphocyte count (7). Many single-cell sequencing analyses have come to similar conclusions, with fewer NK cells in tumors than in normal tissue (8). In fighting solid tumors, NK cells may have an advantage over T cells like enhancing the response to radioimmunotherapy and chemotherapy (9, 10), thus exhibiting important prognostic significance (11–14), despite having less tumor immune infiltration (15).

With the continuous development of bioinformatics, biomarkers have been defined in various ways. NK cell therapy in combination with conventional oncology treatments can effectively improve the outcome of oncology treatment (16). Currently, the function of NRGs in HNSCC is not clear in terms of diagnosis and prognosis. Hence, this study aimed to comprehensively analyze the relationship between the expression pattern of NRGs and the prognosis of HNSCC.

In our study, 17 reliable NRGs were screened to establish a prognostic model *via* TCGA-HNSCC cohort, and we continued to form a risk score to analyze the relationship between NRGs and immune microenvironment, immunotherapy as well as chemotherapy sensitivity. We aim to demonstrate the value of NRGs for assessing the prognosis of HNSCC patients through a comprehensive analysis of genomic data, as well as to develop new tools for improving treatment options.

## Method

### Patient data sources

We downloaded gene expression profiles and clinical data of TCGA-HNSCC cohort including 504 tumor patients and 44 normal controls from TCGA database (https://portal.gdc.cancer.gov/). The level 3 HTSeq-Fragments per kilobase million (FPKM) data of TCGA-HNSCC was converted to TPM (transcripts per million reads) according to the following formula: TPMn = FPKMn * 106/(FPKM0 +… + FPKMm), where n represented gene n and m represented the total number of all genes, respectively. Then, we performed log_2_-based transformation of TPM. The sample size of HNSCC patients at the M stage varied greatly. This stage was consequently excluded from the analysis. Among them, 501 HNSCC samples with complete clinical information were used as the train cohort for further analysis. The gene profiles and clinical data of 97 HNSCC patients in GSE41613 dataset were downloaded from the GEO database (https://www.ncbi.nlm.nih.gov/geo/). The GSE41643 was considered as an external validation dataset.

### Model construction and validation

The immport portal website contains 134 NRGs that are related to NK cells (https://www.immport.org/resource) (**Supplementary Table 1**) and the Molecular Signature Database (MSigDB) covers 18 NK cells associated Gene Ontology (GO) pathways (**Supplementary Table 2**) (17). Finally, 244 NRGs were obtained after eliminating duplicate genes from both datasets. A differential analysis between normal and tumor groups was performed using the “limma” R package (18), based on a screening threshold of |log_2_FC| > 0.5 and an adjusted *P*-value <0.05. By performing univariate Cox regression analysis, we identified genes associated with survival, followed by LASSO regression analysis with “glmnet” in R, and tenfold cross-validation was used to determine the penalty regularization parameter λ. In the following steps, multivariate Cox regression models were used to identify and calculate the coefficients for the central genes. Based on the best lambda values and the corresponding coefficients, we constructed risk signatures based on 17-NRGs. For each patient, the NRGs risk score was calculated as follows, risk score = Expression_mRNA1_ × Coef_mRNA1_ + Expression_mRNA2_ × Coef_mRNA2_ +… Expression_mRNAn_ × Coef_mRNAn_.

### Model formula

All HNSCC patients were given risk scores based on output model equations, and median value were calculated using R package “survminer”, classifying all HNSCC patients into low-risk and high-risk groups, and plotting survival curves for the two subgroups. The R package “pec” was adopted to calculate the C-index. For assessing genetic traits’ predictive power, ROC curve analysis using the “time-ROC” R package was conducted. Decision curve analysis (DCA) of a multi-factor Cox regression model was plotted using the “ggDCA” R package.

### Independent prognostic analysis and nomogram construction

We conducted univariate and multivariate Cox regression analyses to assess risk score as an independent prognostic factor. Using the “rms” R package, histograms were constructed using risk scores versus clinicopathologic characteristics to predict survival at 1, 3, and 5 years for patients in TCGA-HNSCC cohort.

### Functional enrichment analysis

Gene Set Variation Analysis (GSVA) was performed using “c2.cp.kegg.v7.4.symbols.gmt” from the MSigDB. Using “GSVA” R package to perform GSVA enrichment analysis. The “heatmap” R package was used to create heat maps. According to the “limma” R package, an adjusted *P*-value < 0.05 indicates statistical significance for subgroup differences. Through functional enrichment analysis of differentially expressed genes in HNSCC associated with NRGs, functional annotation and enrichment pathways have been explored. The analysis of GO and Kyoto Encyclopedia of Genes and Genomes (KEGG) pathways was done using the “ClusterProfiler” R package, where *P*-value < 0.05 represents a statistically significant difference. The “circlize” R package visualized GO and KEGG results.

### Immunity analysis of the risk signature

Currently accepted methods, including XCELL (19, 20), TIMER (21, 22), QUANTISEQ (21, 22), MCPCOUNT (23), EPIC (24), CIBERSORT (21, 25) and CIBERSORT-ABS (26) were used to measure immune infiltration scores. Spearman correlation analysis was used to examine the correlation between immune cells and risk scores. Based on the immune cell characteristics of HNSCC patients, the single sample GSEA (ssGSEA) method was adopted to differentiate patients at low-risk from those at high-risk. Using a list of 20 suppressive immune checkpoints derived from Auslander’s study, we assessed the suppression of immune checkpoints between high-risk and low-risk groups (27). The “estimate” R package was used to calculate the immunological and mechanistic scores of the specimens from the RNA-seq data to assess the purity of the tumors. Evaluation and visualization of immunotherapy efficacy in HNSCC patients by “limma” and “ggpubr” R package.

Xu et al. developed a website that provided us with gene sets related to cancer and immunity (28) (http://biocc.hrbmu.edu.cn/TIP/) and a set of genes positively associated with anti-PD-L1 drug response was obtained (23) from Mariathasan’s study features (29). Enrichment scores for gene feature positively associated with cancer immune cycles and immunotherapy were calculated between two subgroups by GSVA algorithm (30) and *P*-value <0.05 was considered statistically different. The R package “ggcor” for the analysis of correlations between risk scores and the two genetic traits mentioned above was used.

### Drug sensitivity

The “pRRophetic” R package was used to assess treatment response in high-risk and low-risk groups of patients, as determined by the half-maximal inhibitory concentration (IC50) of each HNSCC patient on the Genomics of Drug Sensitivity in Cancer (GDSC) (https://www.cancerrxgene.org/) (31).

### Gene set cancer analysis database

GSCALite (http://bioinfo.life.hust.edu.cn/web/GSCALite/) provides an online cancer genomic analysis platform by integrating 33 cancers data from TCGA and normal tissue genomics data from GTEx (32). In this study, we analyzed the genomic level, copy number level, methylation level and pathway activity of NRGs in HNSCC by GSCALite.

### Tumor immune single cell hub database

TISCH (http://tisch.comp-genomics.org) is an large-scale online database of single-cell RNA-seg focused on the tumor microenvironment (TME) (33). In this database, the heterogeneity of TME in various cell types and data sets was systematically examined.

### Statistical analysis

Statistical analyses were performed using R software v4.1.3. Kaplan-Meier (KM) survival curves and log-rank test were used to compare OS between high- and low-risk groups. LASSO-Cox regression model was used to construct NRGs signature. Time-dependent ROC was used to evaluate the predictive performance of the model. Spearman correlation analysis was used to evaluate the correlation between risk score and immune cell infiltration. Wilcox test was used to compare the proportion of tumor-infiltrating immune cells, immune checkpoints, and immune function between the two groups. *P*-values <0.05 were considered statistically significant and false discovery rate (FDR)<0.05 was considered statistically significant.

## Result

### Identification of candidate NRGs

**Figure 1:**
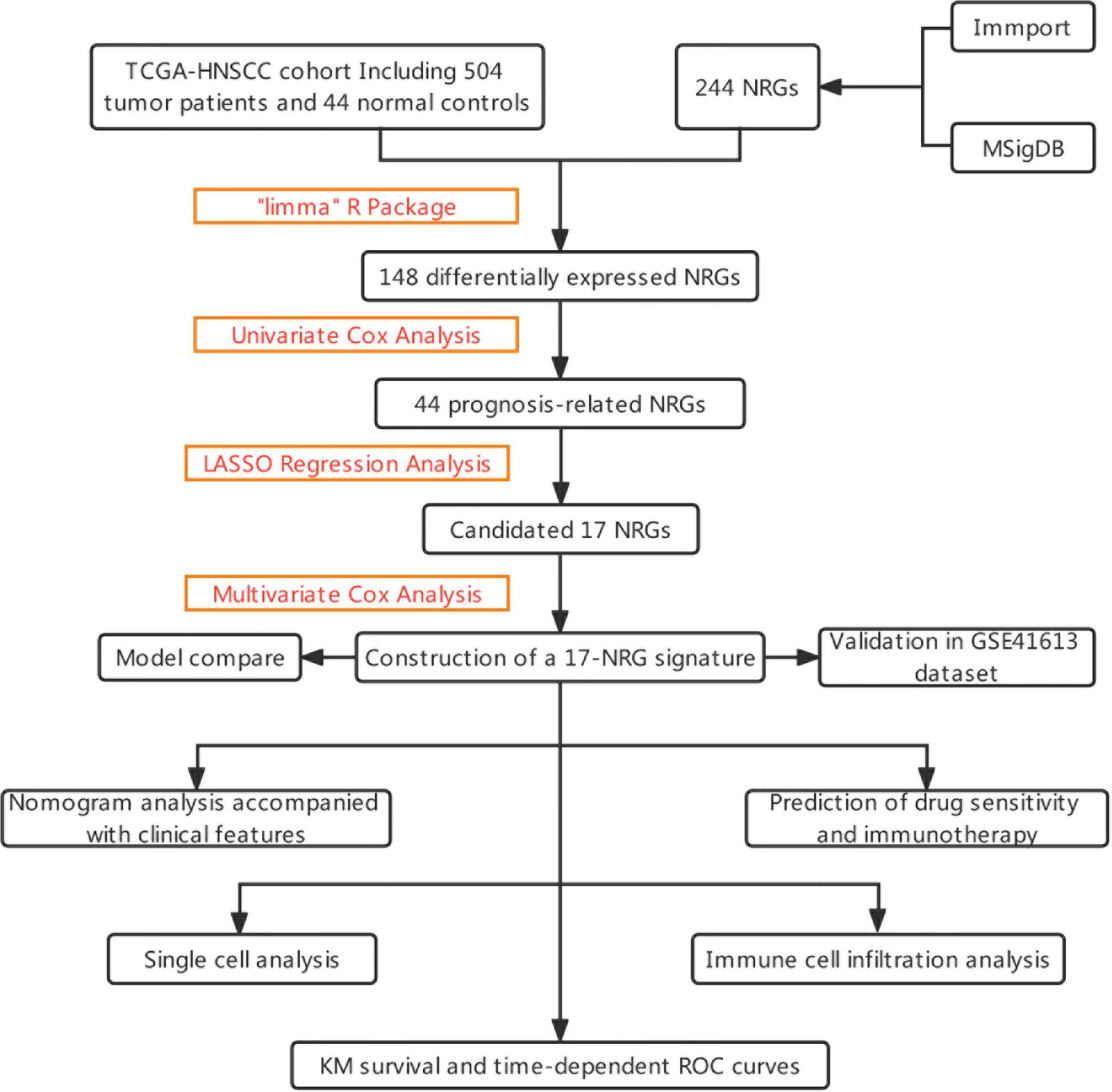
The flowchart summarizes the main design of the present study.

**Figure 2:**
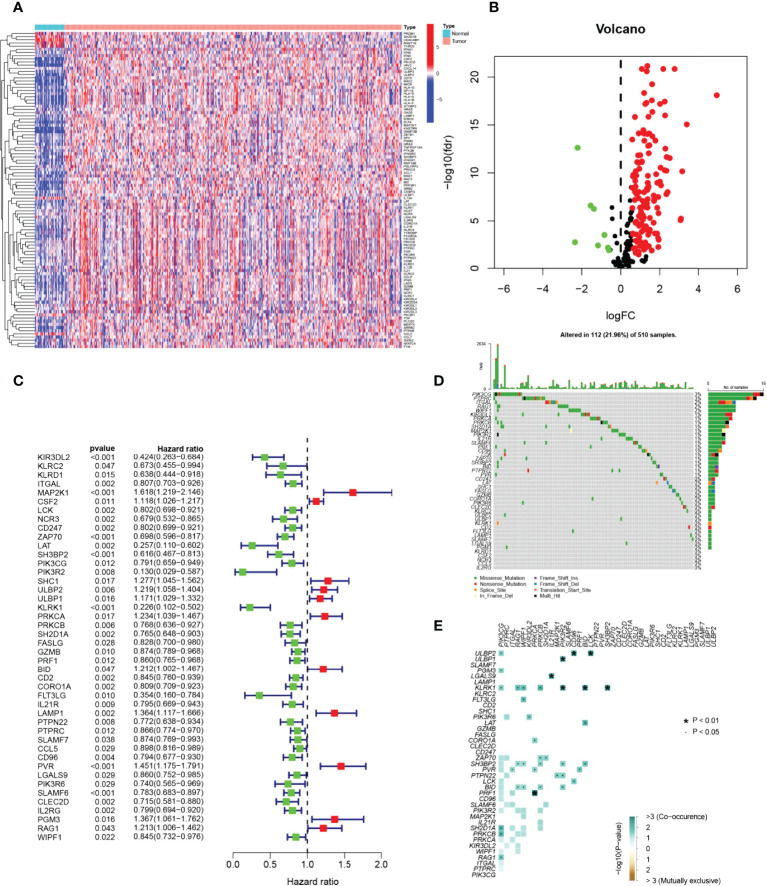
Identification of candidate NRGs. **(A, B)** Heat map and volcano plot of differentially expressed NRGs. **(C)** Prognosis of 44 NRGs in the whole HNSCC cohort analyzed by univariate Cox regression model. **(D)** Oncoplot of 44 mutant NRGs in the HNSCC cohort. **(E)** Co-mutation analysis of NRGs.

The graphical flow chart outlines the main design of this study (**Figure 1**). Using R’s “limma” package, we analyzed gene expression differences in HNSCC patients, and 140 NRGs with up-regulated expression and eight NRGs with down-regulated expression were obtained with |log_2_FC|>0.5. The heat map (**Figure 2A**) and volcano map (**Figure 2B**) were plotted by the “pheatmap” R package. Univariate Cox regression analysis of 148 differentially expressed NRGs (DE-NRGs) identified NRGs associated with OS (*P*<0.05) (**Figure 2C**), and mutation correlation analysis was performed on 501 HNSCC samples. We found that 44 NRGs were mutated on 112 patients with a frequency of 21.96% (**Figure 2D**). Correlation analysis of 44 prognosis-related DE-NRGs was performed by the “maftool” R package, which identified 61 pairs of genes susceptible to co-mutation, of which eight pairs were highly susceptible to co-mutation (**Figure 2E**).

### Construction and validation of NRGs signature

**Figure 3:**
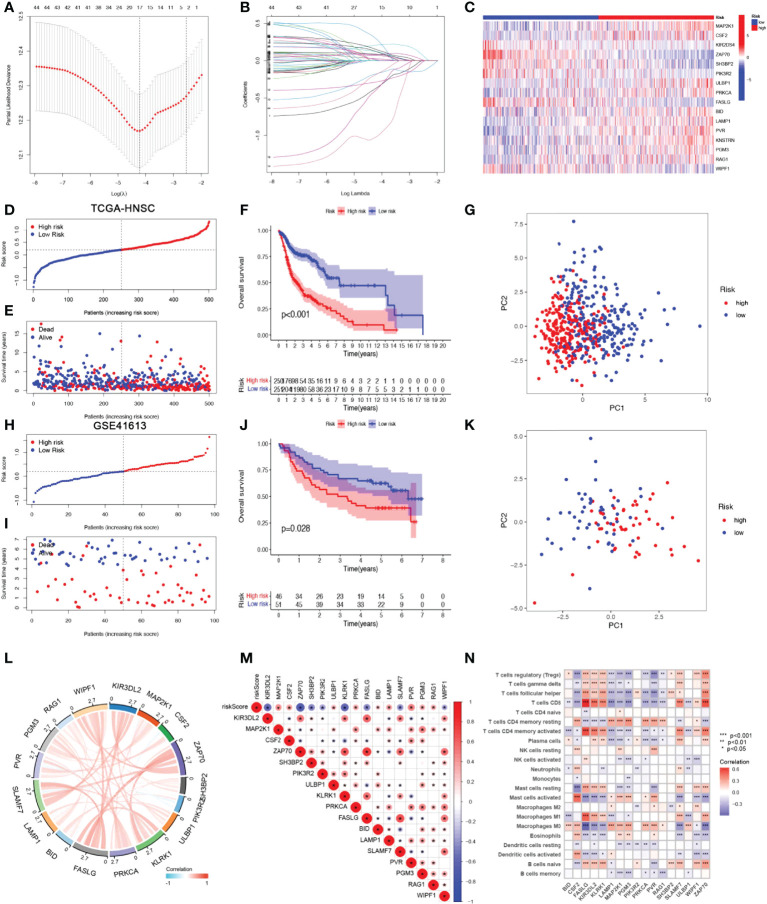
Construction and validation of NRGs Signature. **(A)**Ten‐time cross‐validation for tuning parameter selection in the LASSO model. **(B)**LASSO coefficient profiles. **(C)** Heat map of risk factors in high- and low-risk patients. **(D)** Distribution of risk scores between low- and high-risk groups in the TCGA group. **(E)** Survival status of HNSCC patients in the low- and high-risk groups in the TCGA group.**(F)** (KM curve compares the overall HNSCC patients between low- and high-risk groups in the TCGA cohort. **(G)** PCA plot in the TCGA cohort. **(H)** Distribution of risk scores between low- and high-risk groups in the GEO cohort.**(I)** Survival status of HNSCC patients in the low- and high-risk groups in the GEO cohort. **(J)** KM curve compares the overall HNSCC patients between low- and high-risk groups in the GEO cohort. **(K)** PCA plot in the GEO cohort. **(L)** Correlation analysis and co-expression heat map of 17-NRGs. **(M)** Correlation of 17 NRGs with riskscore **(N)**Correlation analysis of 17 NRGs with immune cells.

A risk score model based on NRGs was developed in order to identify a biomarker that predicts the prognosis of patients with HSNCC. LASSO regression analysis was performed for DE-NRGs with prognostic value, and LASSO regression curves **(** **Figure 3A** **)** and cross-validation plots **(** **Figure 3B** **)** were obtained. The number of genes involved in model construction was obtained by the lowest point of the cross-validation graph as 17, namely KIR3DL2, MAP2K1, CSF2, ZAP70, SH3BP2, PIK3R2, ULBP1, KLRK1, PRKCA, FASLG, BID, LAMP1, SLAMF7, PVR, PGM3, RAG1, and WIPF1. Prognostic index (PI) = (-0.369*KIR3DL2 exp.) + (0.207*MAP2K1 exp.) + (0.026*CSF2 exp.) + (-0.177*ZAP70 exp.) + (-0.286* SH3BP2 exp.) + (-1.020*PIK3R2 exp.) + (0.122*ULBP1 exp.) + (-0.395*KLRK1 exp.) + (0.113*PRKCA exp.) + (0.294*FASLG exp.) + (0.150*BID exp.) + (0.070*LAMP1 exp.) + (0.009*SLAMF7 exp.) + (0.081*PVR exp.) + (0.013*PGM3 exp.) + (0.086*RAG1 exp.) + (-0.187*WIPF1 exp.). On the basis of median scores, we also calculated prognostic risk scores for HNSCC patients. According to the risk heat map **(** **Figure 3C** **)**, high-risk genes correlate positively with risk and low-risk genes correlate negatively. In the TGCA-HNSCC cohort, survival of HNSCC patients showed an increase in mortality with increasing risk (**Figures 3D, E** **)**, and a better prognosis was observed in the low-risk group (*P*<0.001) (**Figure 3F**). Based on principal component analysis (PCA) analysis, low-risk and high-risk patients were clearly separated **(** **Figure 3G** **)**. In the GSE41613 cohort, we demonstrated the same results as in the TCGA-HNSCC cohort. Increasing risk scores increased mortality for patients (**Figures 3H, I** **)**. The KM survival analysis indicated that low-risk patients have an improved prognosis when compared to high-risk patients (P=0.028). **(** **Figure 3J** **)**. PCA analysis suggested significant differences between low- and high-risk patients (**Figure 3K**). Based on the above results, we can conclude that the construct of our prognostic model is quite superior. In addition, the results of correlation analysis for 17-NRGs **(** **Figure 3L** **)** indicated that most of them correlated positively. We then further explored the correlations between these 17-NRGs expressions and risk scores and found that all of these 17-NRGs we studied were closely associated with risk scores. Among them, KIR3DL2, ZAP70, SH3BP2, PIK3R2, KLRK1, FASLG, SLAMF7, and WIPF1 had a significantly negative correlation with risk scores, whereas the rest had a significantly positive correlation with risk scores **(** **Figure 3M** **)**. Meanwhile, to investigate the relationship between the expression of these genes and immune infiltration, we examined the correlation between 17-NRGs and a variety of immune cells and found that 17-NRGs expression was closely associated with various immune cells including NK cell resting and NK cell activated **(** **Figure 3N** **)**.

### Establishment of nomograms in combination with clinical characteristics

**Figure 4:**
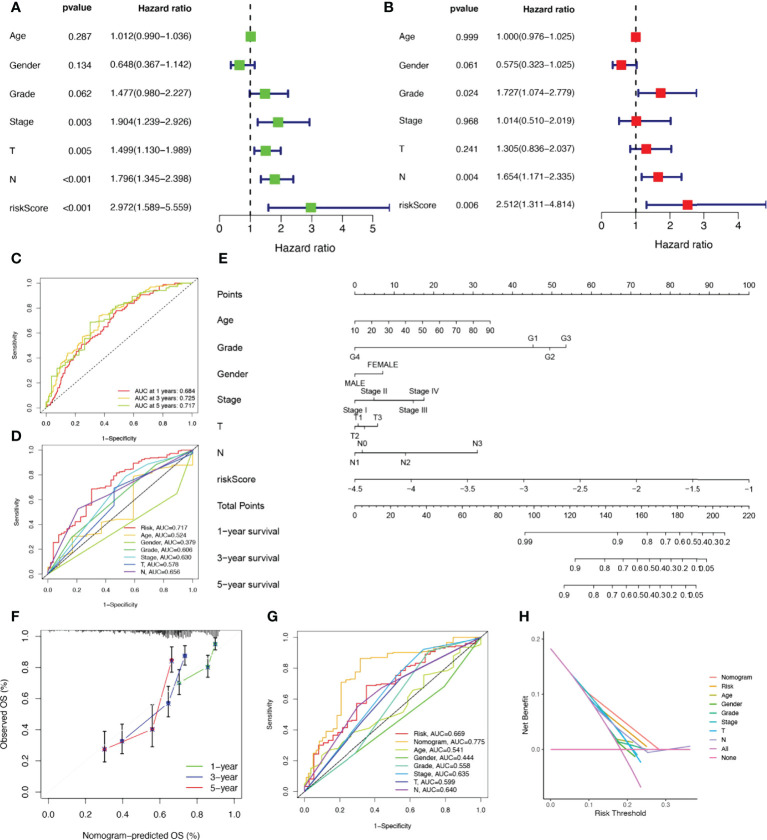
Establishment of nomograms in combination with clinical characteristics. **(A)** Univariate and **(B)** multivariate COX regression analysis of the signature and different clinical feature. **(C)** Time-dependent ROC curves analysis. **(D)** Multi-index ROC analysis **(E)** Nomogram for predicting 1, 3, and 5-year OS of patients with HNSCC. **(F)** The calibration curve of the constructed nomogram of 1, 3, and 5-year survival. **(G)** The nomogram’s time-dependent ROC curves. **(H)** Decision curve analysis.

Considering that the constructed risk model was significantly associated with poor prognosis, we combined the OS of HNSCC patients with their clinical characteristics in univariate and multivariate Cox analyses to determine whether our prognostic characteristics derived from 17 NRGs are independent predictors of survival. Univariate cox analysis revealed that risk score, age, gender, grade, and stage were significantly associated with prognosis in HNSCC (*P*<0.001) (**Figure 4A**). The outcome risk score (P=0.006) was found to be an independent and reliable predictor of outcome risk **(** **Figure 4B** **)**. For HNSCC patients, ROC curves were used to assess the model’s accuracy. **Figure 4C** indicated that the prediction model is highly sensitive and specific depending on the AUC values for risk score, 1, 3, and 5- years (AUC = 0.684, 0.725, 0.717). In addition, the risk score (AUC=0.717) was a better predictor of HNSCC prognosis compared with traditional clinicopathological features (**Figure 4D**). To enlarge the clinical application and usability of the constructed risk model, we constructed a nomogram based on gender, age, stage, grade, and risk score to predict the probability of prognostic survival at 1, 3, and 5 years in HNSCC patients (**Figure 4E**). The risk score was found to have the greatest impact on predicting OS, showing that HNSCC could be more accurately predicted when using a risk model based on 17 NRGs. The calibration curves also showed satisfactory agreement between predicted and observed values in terms of the probability of 1, 3, and 5-year OS (**Figure 4F**). Nomogram showed the best predictive power (AUC=0.775), compared to risk (AUC=0.717) and clinical characteristics (**Figure 4G**). Multiple results demonstrated that our model had the highest net benefit, suggesting that risk models constructed based on NRGs are more influential than traditional models in clinical decision-making (**Figure 4H**).

### Correlation analysis of clinicopathological characteristics and risk scores

To analyze the association between high and low-risk groups and clinical characteristics, heat maps were drawn based on clinical characteristics, risk score, and the expression of 17-NRGs, showing the association between 17-NRGs identified in the prognostic risk model and the clinical characteristics and risk scores of all HNSCC patients in TCGA (**Supplementary Figure 1A**). The distribution of tumor stage, grading, T stage, and N stage was significantly different among high and low-risk groups, while in both subgroups, neither age nor gender differed significantly (**Supplementary Figure 1B**). We then used the Wilcoxon test to compare the differences in risk scores of clinical characteristics among the subgroups and thus test the correlation between them. Results revealed a significant association between risk scores and tumor pathological stage, grade, and T stage (*P*<0.05) (**Supplementary Figures 1C–H**).

### Clinical subgroup survival analysis of the NRGs signature

**Figure 5:**
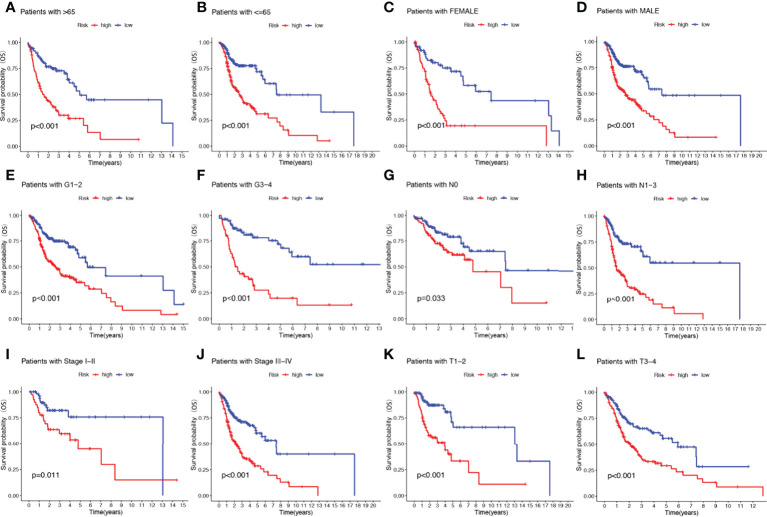
The risk score based on 17-NRGs signature is a valuable marker for poor prognosis in various subgroups divided by clinicopathological characteristics. The NRGs could distinguish high-risk patients in a variety of subgroups divided by clinicopathological characteristics including age **(A, B)**, gender **(C, D)**, grades **(E, F)**, N stage **(G, H)**, tumor stage **(I, J)** and T stage **(K, L)**.

To further understand whether the prognosis of patients in different clinical subgroups differed, clinical analysis of the entire sample subgroup was performed. All samples were divided into different subgroups according to age (>65 years and ≤65 years), gender (male and female), tumor grade (grades I-II and III-IV), pathological N stage (N0 and N1-3), T stage (T1-2 and T3-4), and pathological stage (I-II and III-IV) for further survival analysis. The survival time of high-risk patients in all subgroups was significantly shorter than that of low-risk patients **(** **Figure 5** **)**. It appears that the NRGs risk model currently identified can also reliably predict the prognosis of certain subgroups of HNSCC based on their clinical characteristics.

### NRGs signature performed better than others in prognostic prediction

To further demonstrate whether our constructed 17-NRGs signature has the accurate predictive capability for HNSCC patients, we compared it with five published prognostic signatures, namely the Wang signature (34), Wu signature (35), Xue signature (36), Yang signature (37) and Zhu signature (4). For comparability of signatures, we calculated the risk score of each HNSCC sample in entire TCGA cohorts by the same method and converted the risk score according to the previous methods in the five signatures. Although the five signatures effectively divided HNSCC patients into two subgroups with significantly different prognoses, they exhibited lower AUC values than our model at 1, 3, and 5-year survival (**Supplementary Figures 2A-F**). Furthermore, the RMS analysis and C-index analysis also show that our signature performs significantly better than the other signature (**Supplementary Figures 2G, H** **)**. All these results clearly indicated that the constructed 17-NRGs signature performs exceptionally well in terms of predictive capability.

### Functional enrichment analysis

**Figure 6:**
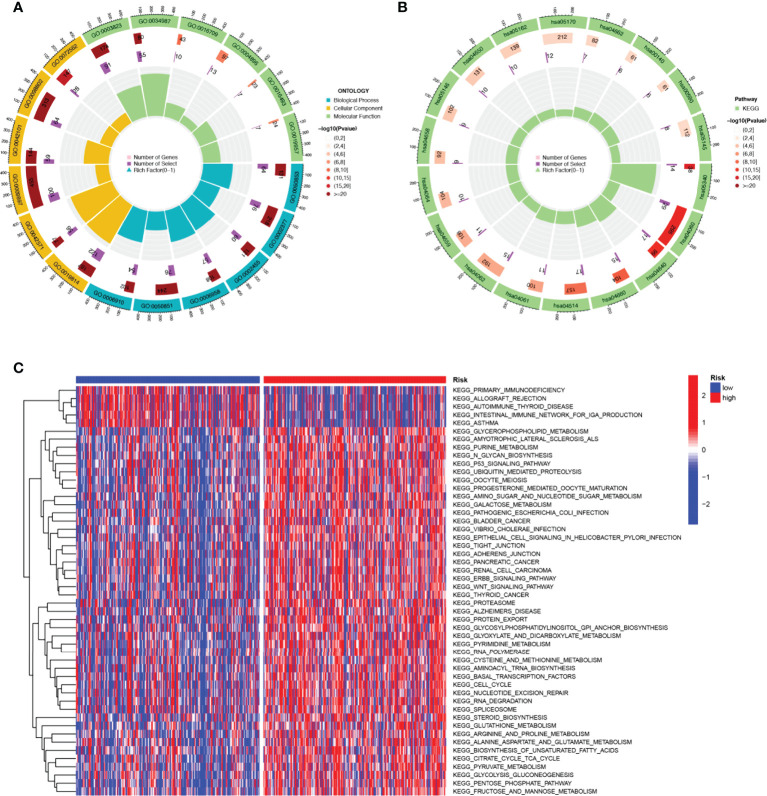
The enriched items in functional analysis. **(A)** GO enrichment analysis. **(B)** KEGG enrichment analysis. **(C)** GSVA analysis between the high-risk cohort and the low-risk cohort.

KEGG enrichment analysis and GO functional analysis were performed to assess differential genes between the two subgroups to elucidate the relevance of bioactivity and signaling pathways to risk scores. The threshold FDR<0.05 and *P*<0.05 were used to select significantly enriched items. Biological processes (BP) mainly included immunoglobulin production, B cell receptor signaling pathway, humoral immune response mediated by circulating immunoglobulin and antigen receptor-mediated signaling pathway, etc. The cellular component (CC) mainly included the external side of the immunoglobulin complex, T cell receptor complex, and plasma membrane signaling receptor complex. Molecular function (MF) mainly included immunoglobulin receptor binding, cytokine receptor activity, and antigen binding (**Figure 6A** **;** **Supplementary Table 3**). KEGG mainly included PD-1 checkpoint pathway, T cell receptor signaling pathway,natural killer cell mediated cytotoxicity, PD-L1 expression, and Cytokine-cytokine receptor interaction in cancer (**Figure 6B** **;** **Supplementary Table 4**). GSVA analysis identified 111 significantly enriched pathways (**Figure 6C** **;** **Supplementary Table 5**), and among low-risk individuals, pathway enrichment mostly involved immune function, including primary immunodeficiency, allograft rejection, etc. In summary, we were surprised to find a strong correlation between enrichment analysis results and immune response, and therefore we conducted a systematic analysis of the immune landscape in the two subgroups of HNSCC patients.

### NRGs risk score predicts immune cell infiltration and TME

**Figure 7:**
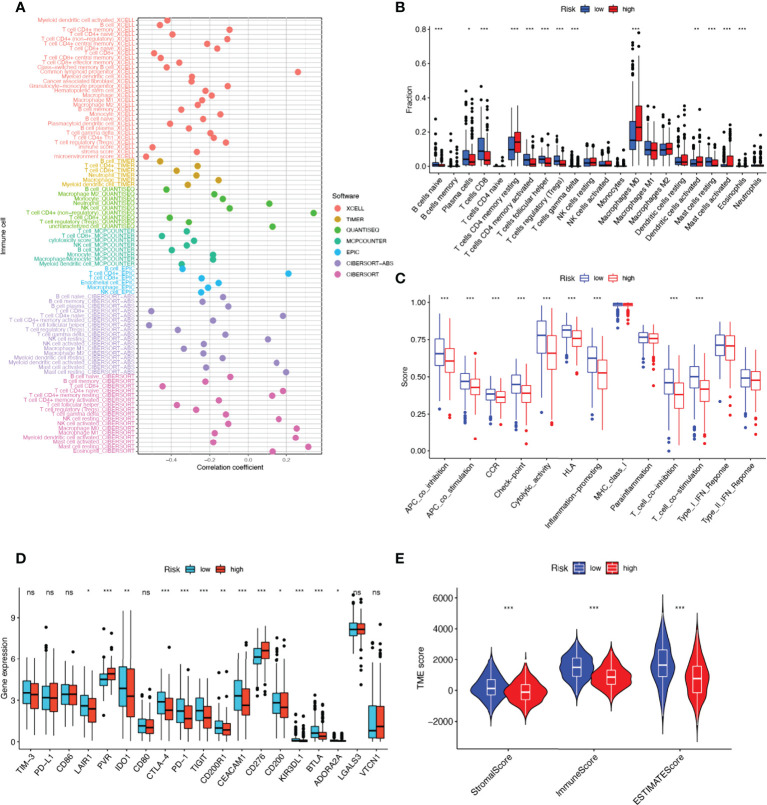
17-NRGs risk score predicts tumor microenvironment and immune cell infiltration. **(A)** Immune cell bubble map. **(B)** Differences in immune cell infiltration between high and low-risk groups. **(C)** Immune function ssGSEA scores between high and low-risk groups. **(D)** Immune checkpoint differences between high- and low-risk groups. **(E)** TME component analysis. *P < 0.05, **P < 0.01, ***P < 0.001, ns ≥ 0.05.

Tumor immune cell infiltration is widely considered to be one of the important immune features of TME. Spearman correlation analysis was performed to find a correlation between the risk score and the abundance of immune cells in the HNSCC tumor microenvironment *via* various algorithms. For NK cell infiltration, QUANTISEQ, MCPCOUNTER, and EPIC showed negative correlations with risk scores. It is interesting to note that, in CIBERSORT-ABS and CIBERSORT results, NK cells resting had a positive correlation with risk scores, but NK cells activated had a negative correlation with risk scores **(** **Figure 7A** **)**. To understand the distribution and correlation of the relative content of 22 TICs (tumor-infiltrating immune cells) in the TCGA-HNSCC cohort, we calculated the level of immune cell infiltration in each sample by the CIBERSORT algorithm. As compared with the high-risk group, the low-risk group appeared to have higher levels of immune infiltration, except for activated dendritic cells, CD4 memory resting T cells, and M0 macrophages **(** **Figure 7B** **)**. According to the results, NRGs risk score models are capable of classifying different immune subtypes and thus influence the response to immunotherapy. Differences in immune cell infiltration may lead to alterations in immune function, so we performed single sample GSEA (ssGSEA) score comparison of immune function, and the vast majority of immune function scores were significantly greater in the low-risk group versus high-risk group **(** **Figure 7C** **)**. Further, we explored the differences in immune checkpoint expression between the two groups, due to the importance of immune checkpoints for the effectiveness of immunotherapy in tumors. Low-risk individuals showed significant upregulation of 11 immune checkpoint genes, including LAIR1, IDO1, CD200R1, CEACAM1, CD200, KIR3DL1, BTLA, ADORA2A, CTLA-4, PD-1, TIGIT. While high-risk group showed significant upregulation of PVR and CD276 immune checkpoint genes **(** **Figure 7D** **)**. Upregulation of immune checkpoint is a key feature of inflamed TME (38) and may suggest that low-risk patients are in an inflammatory microenvironment. Targeted therapy against immune checkpoints with elevated expression could potentially benefit patients with this subtype of tumor (39–41). Subsequently, stromal score, immune score, and ESTIMATE score were higher in the low-risk group (*P*<0.001), indicating the higher overall immune level and immunogenicity of the TME in the low-risk group **(** **Figure 7E** **)**.

### NRGs risk score predicts treatment response assessment

**Figure 8:**
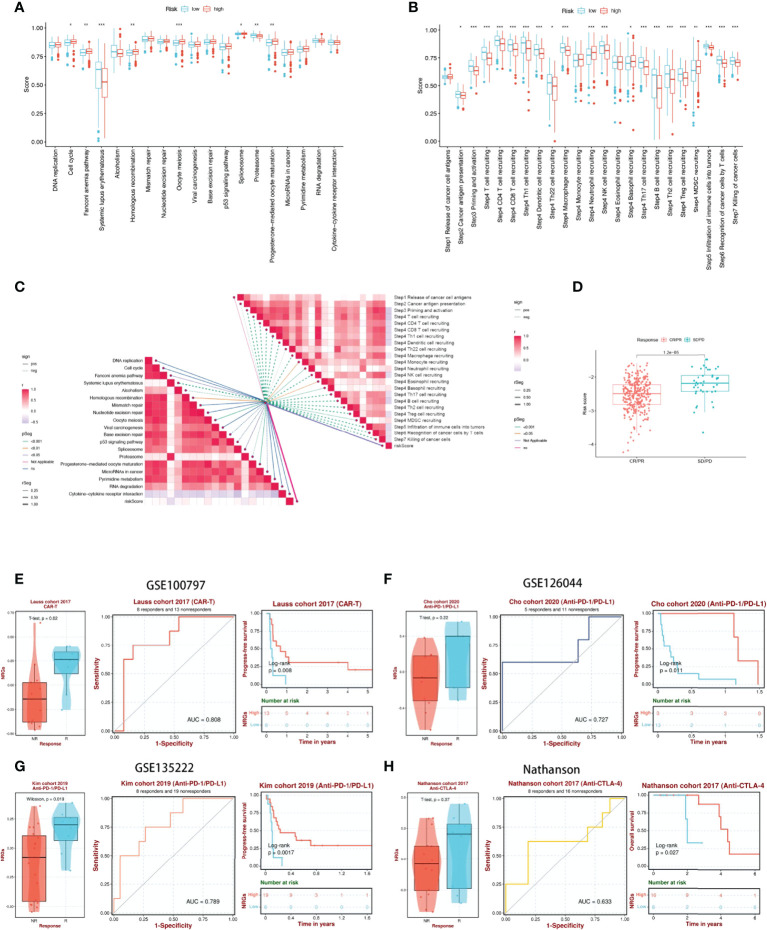
17-NRGs risk scores predicting treatment response assessment. **(A)** The plot of the difference in enrichment scores between the high-risk and low-risk groups on the immunotherapy prediction pathway. **(B)** The plot of differences between the high-risk and low-risk groups on each step of the cancer-immune cycle. **(C)** Correlation of risk scores with ICB response signature and each step of the tumor-immune cycle. **(D)** Correlation between risk scores and clinical response to cancer immunotherapy. **(E)** Evaluation of NRGs for CAR-T therapy in the GSE100797 cohort. **(F, G)** Evaluation of NRGs for anti-PD-L1 therapy in the GSE126044 and GSE135222 cohorts. **(H)** Evaluation of NRGs for anti-CTLA-4 treatment in the Nathanson cohort. PD: disease progression; SD: stable disease; PR: partial response; CR: complete response. TIDE, Tumor Immune Dysfunction, and Exclusion. *P < 0.05, **P < 0.01, ***P < 0.001.

We first analyzed the differences in predicting immune checkpoint blockade (ICB) response signatures between the two subgroups. The low-risk group scored highly for Systemic lupus erythematosus and Proteasome, while scored lowly for Oocyte meiosis, Progesterone-mediated oocyte maturation, Cell cycle, Spliceosome, Fanconi anemia pathway, and Homologous recombination than those of the high-risk group **(** **Figure 8A** **)**. The correlation between NRGs risk scores and ICB-related positive signals was further explored subsequently. The results showed that risk scores were associated with Fanconi anemia pathway, Homologous recombination, Oocyte meiosis, Spliceosome, Progesterone-mediated oocyte maturation were significantly positively correlated with Systemic lupus erythematosus, Alcoholism, and Proteasome **(** **Figure 8C** **)**. The tumor immune cycle is a key indicator to evaluate the biological function of the chemokine system and other immunomodulators (28, 42). Therefore, we analyzed the differences in the activity of tumor immune steps between high and low-risk groups, and in low-risk group, an upregulation of the activity of most steps of the cycle was observed, including cancer cell antigen expression (step 2), initiation and activation (step 3), immune cell trafficking into tumors (step 4) (T cell recruitment, CD4 T cell recruitment, CD8 T cell recruitment, Th1 recruitment, DC cell recruitment, Th22 cell recruitment, macrophage recruitment, NK cell recruitment, Th17 cell recruitment, B cell recruitment, Th2 cell recruitment, Treg cell recruitment and), Infiltration of immune cells into tumors (step 5), Recognition of cancer cells by T cells (step 6), Killing of cancer cells (step 7). While, neutrophil recruitment (step 4), Basophil recruitment (step 4), MDSC recruitment (step 4) activity were decreased **(** **Figure 8B** **)**. Similarly, we investigated the correlation between these steps in the tumor immune cycle and risk scores. In step 4, a significant positive correlation was found between risk score and monocyte recruitment, neutrophil recruitment, Eosinophil recruitment, and Basophil recruitment, while significantly negatively correlated with each of the remaining tumor immune cycle steps **(** **Figure 8C** **)**. More importantly, 17-NRG expression was significantly higher in patients with progressive and stable disease than in those with partial or complete responses (P=1.2e-05) **(** **Figure 8D** **)**, suggesting that patients with higher NRGs expression might respond worse to ICB. Based on the BEST database, we examined four external cohorts (GSE100797, GSE126044, GSE135222, Nathanson) receiving immunotherapy for the association between NRGs scores and the benefits of immunotherapy (https://rookieutopia.com/). High NRGs expression in patients showed a higher degree of immune response to CAR-T, anti-PD-L1, and anti-CTLA-4, and the ROC curves confirmed the efficacy of NRGs in predicting immunotherapy responsiveness **(** **Figures 8E-H** **)**.

### NRGs signature predicts chemotherapy sensitivity

**Figure 9:**
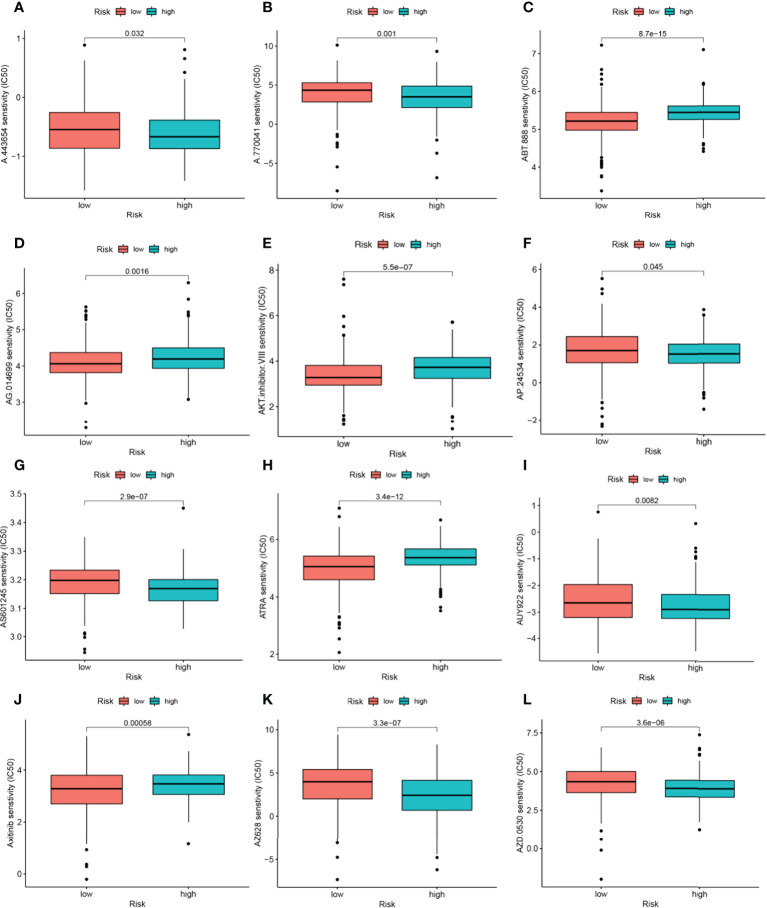
17-NRGs signature predicts chemotherapy sensitivity. **(A)** A.443654, **(B)** A.770041, **(C)** ABT.888, **(D)** AG.014699, **(E)** AKT.inhibitor. VIII, **(F)** AP.24534, **(G)** AS601245, **(H)** ATRA, **(I)** AUY922, **(J)** Axitinib, **(K)** AZ628, **(L)** AZD.0530.

Among the 12 immunotherapeutic agents applied in the treatment of HNSCC, the low-risk group included A.443654 (*P*=0.032), A.770041 (*P*=0.001), AP.24534 (*P*=0.045) AS601245 (*P*=2.9e-07), AUY922 (*P*=0.0082), AZ628 (*P*=3.3e-07), AZD.0530(*P*=3.6e-06). IC50 was relatively high compared to the high-risk group **(** **Figures 9A, B, F, G, I, K, L** **)**. In addition, we found five other chemical or targeted drugs ABT.888 (*P*=8.7e-15), AG.014699 (*P*=0.0016), AKT.inhibitor.VIII (*P*=5.5e-07), ATRA (*P*=3.4e-12), and Axitinib (*P*=0.00058), with lower IC50 in the low-risk group lower **(** **Figures 9C, D, E, H, J** **)**. Based on the risk score, we could further investigate the immunotherapy response in HNSCC patients and enhance the precise drug treatment.

### Multi-omics mutation analysis of NRGs

Furthermore, we examined different histological levels, including genomics and copy number, to understand the biological mechanism of NRG aberrant expression. The single nucleotide locus variation (SNV) results showed that Missense_Mutation of NRGs was the most common variant classification in the TCGA-HNSC cohort, while the most prevalent variant type was single nucleotide polymorphism. C>T had the highest frequency compared to other SNV categories **(** **Supplementary Figure 3A** **)**. And mutations occurred in 49 HNSCC patients, with KIR3DL2 possessing the highest mutation frequency **(** **Supplementary Figure 3B** **)**. Subsequently, copy number variation analysis was performed to summarize the ratio of Homozygous mutations and heterozygous mutations in NRGs in the sample **(** **Supplementary Figure 3C** **)**. In addition, Spearman’s correlation coefficient analysis between copy number variants and gene expression was performed, and it was found that copy number variants of KIRK1 and FASLG were down-regulated in HNSCC, while LAMP1, SH3BP2, MAP2K1, PGM3, PIK3R2, BID, ULBP1, PVR, and KIR3DL2 were up-regulated **(** **Supplementary Figure 3D** **)**. We counted the two mutations separately, and the results showed that the samples amplified mainly in heterozygous mutations such as KIRK1, FASLG, SLAMF7, etc., while CSF2, SH3BP2, etc. were copy number deletions. The Homozygous mutations amplified mainly WIPF1, while SH3BP2 showed mainly copy number reduction **(** **Supplementary Figures 3E, F** **)**, so the abnormal gene expression may be the result of both copy number variation and single nucleotide variation. Following this, we investigated the relationship between the activity of cancer-related pathways and the expression of NRGs. Results showed that cell cycle, P13KAKT, RASMAPK, and TSCmTOR pathways were inhibited in HNSCC patients under the regulation of NRGs, while Hormone ER, Hormone AR, Apoptosis, EMT, DNA damage, RTK pathways were activated or inhibited **(** **Supplementary Figure 3G** **).** In addition, we explored that the differential expression of NRGs was significantly correlated with drug sensitivity in the Cancer Therapeutics Response Portal and GDSC databases, respectively **(** **Supplementary Figures 3H, I** **)**. This implies that the expression of our risk profile genes could be used as a predictor of chemotherapy drug sensitivity in patients or could be used as a future drug sensitization target.

### Correlation analysis of NRGs and immune microenvironment

We used the single cell dataset HNSCC_GSE139324 from the TISCH database to analyze the expression of 17-NRGs in the immune microenvironment. In the GSE103322 dataset, there are 23 cell populations and 11 species of immune cell types (**Supplementary Figures 4A, B** **)**, and the distribution and number of various cell types are shown (**Supplementary Figures 4C, D** **)**. The expression levels of each NRGs in immune cells are shown in **Supplementary Figure 4E**. BID, LAMP1, MAP2K1, SH3BP2, WIPF1 and ZAP70 were expressed on various immune cells, while CSF2, KIR3DL2, KLRK1, PGM3, PIK3R2, PVR, RAG1 and ULBP1 were barely expressed in the immune microenvironment. FASLG is mainly expressed in NK, CD8Tex and Tprolif. PRKCA is mainly expressed in CD8T and CD4Tconv. SLAMF7 is mainly expressed in NK, CD8Tex, Tprolif and Mono/Macro.

## Discussion

HNSCC exhibits considerable heterogeneity in terms of human biologic behavior and treatment response, and despite a plethora of therapies, the 5-year overall survival rate for patients with HNSCC remains <50% (43–45). Despite ongoing efforts to develop new concepts of HNSCC in precision medicine, particularly ICB and targeting therapies, over the past few decades, survival hasn’t improved (46). Most patients present with advanced disease when they are diagnosed (47). However, risk stratification by tumor size, lymph node and distant metastasis and histological grade alone is not sufficient to predict prognosis in patients with HNSCC, more accurate models for predicting prognosis are urgently needed (48, 49). NK cells play a crucial role in the tumor microenvironment and immune surveillance, and their associated genes are gaining attention (50). However, a comprehensive analysis of NRGs in HNSCC has not been reported. Therefore, we used mRNA expression data from the TCGA-HNSCC dataset to identify important prognostic genes and developed a multi-biomarker prognostic model based on natural killer cell-associated genes.

In this study, we integrated NRG gene expression profiles from the TCGA-HNSCC dataset and selected 17 genes to construct a new prognostic model for NRGs using LASSO regression analysis and COX risk regression analysis. The NRGs signature we constructed was shown to be an independent prognostic factor for HNSCC and was divided into two different prognostic subgroups based on median risk scores. ROC curves, nomogram, and calibration curves were subsequently constructed, and a comprehensive analysis demonstrated a more prominent predictive performance of HNSCC signature compared to other traditional clinical indicators such as age, gender, histological stage, and tumor grade. At the same time, the predicted values are in satisfactory agreement with the observed values. It can provide a theoretical basis for clinicians’ decision-making.

KIR3DL2, a member of the killer cell immunoglobulin-like receptor, is involved in blocking NK cell activation and function upon contact with HLA-A3 or HLA-A11, resulting in tolerance (51–53). MAP2K1 is involved in regulating mitogen-activated protein kinase (MAPK)-mediated release of cellular granules to target cells, thereby altering the cytotoxic function of NK cells (54, 55). ZAP70 is one of the intracellular kinases that transmit signals upon NK cell binding to target cells, ultimately leading to NK cell activation. It has been shown that *in vitro* NK cell populations with high levels of ZAP70 are more cytolytic compared to those with low levels of ZAZ70 (56). It has been shown that CD244 induces overexpression of SH3BP2, accompanied by increased cytotoxicity in NK cells (57). SH3BP2 may mobilize key downstream signaling effectors to regulate NK cell-mediated cytotoxicity (58). KLRK1 is a receptor expressed by NK cells and cytotoxic T lymphocytes; binds non-covalently to DAP10 signaling protein to provide co-stimulatory or activation signals to T and NK cells (59, 60). Both FASLG and KLRC4-KLRK1 are involved in the apoptosis of NK and T cells and cytotoxicity (61, 62). SLAMF7 homologous interactions regulate NK cell cytolytic activity. SLAMF7 on NK cells can bind to elotuzumab, thereby inducing NK cell activation and enhancing cytolytic function against myeloma cells (63). PVR can be bound by TIGIT and DNAM-1 thereby inhibiting NK cytotoxicity to prevent NK cell self-destruction of normal cells (64). Recombination-activated genes (RAGs) confer the ability to assemble diverse antigen receptor genes by adaptive immune cells. Mutant NK cells lacking RAG (or RAG activity) are more differentiated and cytolytic than NK cells with RAG expression (65). In 2012, the first case of autosomal recessive immunodeficiency caused by a mutation in the WIPF1 gene (resulting in WIP deficiency) was reported in an infant with reduced NK cell function (66). However, the effect of WIPF1 on NK cells remains unclear as to the mechanism of action of WIPF1 on NK cells.

However, significant intra- and inter-tumor heterogeneity is one of the strongest features of HNSCC and hinders the identification of specific biomarkers and the establishment of targeted therapies for the disease (46). Therefore, an “ideal” preclinical cancer model should consider both TME and tumor heterogeneity. Since immune cells are the cellular basis of immunotherapy, an in-depth understanding of immune infiltration in TME is essential to unravel the underlying molecular mechanisms and provide new immunotherapeutic strategies to improve clinical outcomes (67). Therefore, we analyzed immune cell infiltration and immune function expression in high and low-risk groups. In addition, CD8+ T cells were also highly infiltrated in the low-risk group, resulting in a better prognosis for HNSCC patients. Interestingly, cetuximab-activated NK cells were able to promote CD8+ T-cell activation and thus the antitumor immune response in HNSCC (68). Therefore, the use of cetuximab in the low-risk group may have unexpected therapeutic effects by further activating CD8+ T cells.

Immune checkpoints are of interest as one of the important features of TME. Among them, programmed death ligand 1 (PD-L1), an immune checkpoint protein in the cancer-immune cycle, is expressed on the surface of tumor cells (TC) and tumor-infiltrating immune cells (IC) to downregulate T cell function (69). The high expression in the low-risk group may indicate that tumor cells in low-risk patients rely on the PD-1/PD-L1 signaling pathway to evade immune surveillance, and the risk group patients with PD-1 monoclonal antibodies may have good efficacy. In addition, we found that PVR and CD276 expression was higher in the high-risk group, and both have been found to be associated with poor prognosis. PVR modulates NK cells and regulates T-cell activity leading to immunosuppression (70, 71), while CD276 enables HNSCC stem cells to evade immunosurveillance (72). Therefore, patients in the high-risk group need to practice combinations of immunotherapies will become important (73) with the potential to further improve the efficacy of immune checkpoint blockade therapies and expand the population benefiting from immunotherapy. Targeted therapy against immune checkpoints with elevated expression may achieve better results.

Although our study has greater clinical implications for the prognostic assessment and selection of treatment options for patients with HNSCC, our study still has some limitations. First, our study is a retrospective study that needs to be validated in future prospective studies. The potential of this signature to predict the response to immunotherapy was indirectly assessed because mRNA expression profile data were not available for HNSCC patients receiving immunotherapy, which may lead to deviations from the actual situation. Therefore, future validation should be performed in combination with data from HNSCC patients receiving immunotherapy.

## Data availability statement

The original contributions presented in the study are included in the article/**Supplementary Material**. Further inquiries can be directed to the corresponding authors.

## Author contributions

HC and ZX conceived the study. HC, XX, YY, GP and SZ drafted the manuscript. HC and DS performed the literature search and collected the data. HC, XX, GP, GL and SZ analyzed and visualized the data. HC, GL and GT helped with the final revision of this manuscript. All authors contributed to the article and approved the submitted version.

## Funding

This study was supported by a grant from Southwest Medical University (No.2020XSJG-C01-21).

## Acknowledgments

We thank Southwest Medical University for its support of the Student Innovation and Entrepreneurship Program. Special thanks to Zaoqu Liu (The First Affiliated Hospital of Zhengzhou University), and all the members of his team, RookieUtopia, for developing the BEST application (https://rookieutopia.com/).

## Conflict of interest

The authors declare that the research was conducted in the absence of any commercial or financial relationships that could be construed as a potential conflict of interest.

## Publisher’s note

All claims expressed in this article are solely those of the authors and do not necessarily represent those of their affiliated organizations, or those of the publisher, the editors and the reviewers. Any product that may be evaluated in this article, or claim that may be made by its manufacturer, is not guaranteed or endorsed by the publisher.
