# Supplementary material for: Natural killer cell-related prognosis signature characterizes immune landscape and predicts prognosis of HNSCC
Source: Front Immunol. 2022 Oct 3;13:1018685. doi: 10.3389/fimmu.2022.1018685 (PMC9575041; doi:10.3389/fimmu.2022.1018685)
Supplement: Supplementary file 1 [file DataSheet_1.docx]

Supplementary Material

## Supplementary Figures


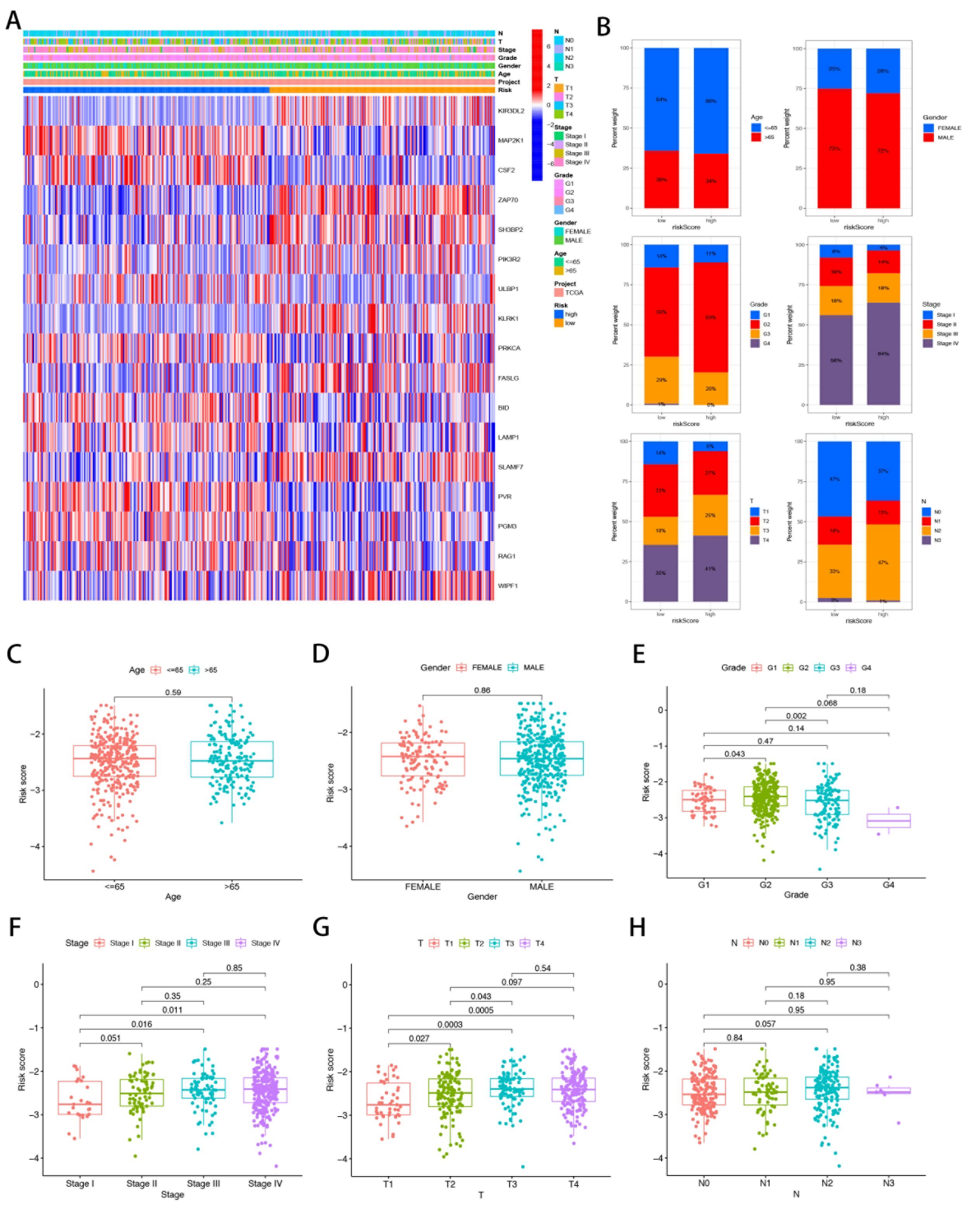


**Supplementary Figure 1.** Correlation analysis of risk scores and clinicopathological characteristics. (**A**) Heatmap for the 17 NRGs-based signature with clinicopathological manifestations. (**B**) Risk score distribution stratified by age, gender, grade, tumor stage, T stage and N stage. The distribution of risk scores according to (**C**) age,(**D**) gender,(**E**) grade, (**F**) tumor stage,(**G**) T stage, and (**H**) N stage.





**Supplementary Figure 2.** Comparison of the NRGs risk model with other models (**A**) KM curves and ROCs for NRGs signature. (**B-F**) KM curves and ROCs for risk models constructed by others (**G**) C-indexes for six risk models. (**H**) RMSs for six risk models.





**Supplementary Figure 3.** Multi-omics mutation characteristics of NRGs. **(A, B)** Classification of mutations in NRGs in HNSCC and their mutation incidence. **(C)** The proportion of different types of copy number variants in NRGs. **(D)** Correlation analysis of copy number variants of NRGs with expression. **(E, F)** Distribution of copy number variants amplification and deletion of NRGs in Homozygous mutation and heterozygous mutations. **(G)** Analysis of the role of NRGs' expression activity in the regulation of cancer-related pathways **(H, I)** Correlation analysis of NRGs' expression and sensitivity to chemotherapeutic drugs in CDRP and CDSC cohorts.


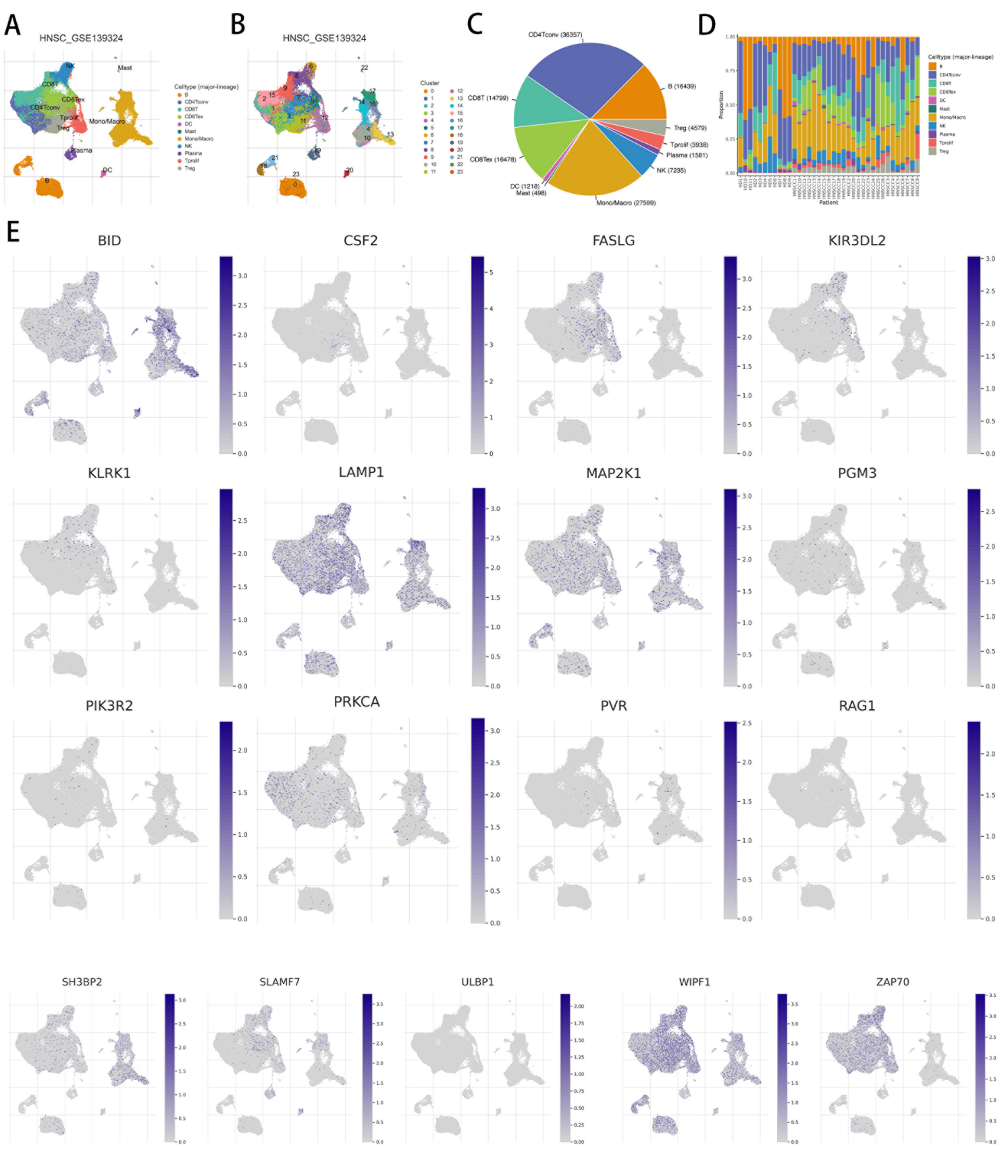


**Supplementary Figure 4.** NRGs Expression in HNSC TME-associated cells. (**A-D**) Annotation of all cell types in GSE139324 and the percentage of each cell type. (**E**) Percentages and expressions of 17 NRGs.
